# Comprehensive analyses of single-cell and bulk RNA-seq reveal the biological and prognostic roles of *BMP4* in pancreatic adenocarcinoma

**DOI:** 10.3389/fmolb.2025.1686938

**Published:** 2025-10-15

**Authors:** Ao Wang, Xinglong Shen, Shan Lu, Chao Bi, Yang Cao, Yunfeng Liu, Meng Li, Yun Zhao, Huaizhang Jin, Xiaopeng Shen, Wei Dai, Yang Lei

**Affiliations:** ^1^ College of Physical Education, Anhui Normal University, Wuhu, Anhui, China; ^2^ College of Life Sciences, Anhui Normal University, Wuhu, Anhui, China; ^3^ Department of Cadre respiratory, Jinling Hospital, Affiliated Hospital of Medical School, Nanjing University, Nanjing, Jiangsu, China; ^4^ Wuhu Center for Disease Control and Prevention, Wuhu, Anhui, China

**Keywords:** BMP4, PAAD, pan-cancer analysis, ScRNA-seq, prognosis

## Abstract

Bone Morphogenetic Protein 4 (*BMP4*) plays a critical role in development, but its function in pancreatic adenocarcinoma (PAAD) is not well understood. We found that *BMP4* is minimally expressed in the normal pancreas but markedly upregulated in PAAD, correlating with poor patient survival. Pan-cancer analysis revealed distinct expression and prognostic patterns of *BMP4*, while pathway analyses indicated that *BMP4* predominantly regulates metabolic rather than canonical BMP signaling. Single-cell RNA-seq showed *BMP4* enrichment in cancer cells and cancer stem cells, supporting its role in tumor metabolism. Importantly, *BMP4* was identified as an independent prognostic factor for PAAD, and a nomogram incorporating *BMP4* accurately predicted patient outcomes. Although *BMP4* affected certain immune cell infiltrations, its prognostic impact was largely independent of immune modulation. Collectively, these findings highlight *BMP4* as a potential biomarker and therapeutic target in PAAD.

## Introduction

Pancreatic cancer poses a substantial threat to human health, exhibiting the highest mortality rates and the lowest survival rates among all cancer types. It has two main subtypes: pancreatic adenocarcinoma (PAAD) and pancreatic neuroendocrine tumor, with PAAD accounting for over 90% of cases. The prognosis for PAAD remains grim, with less than 13% of patients surviving 5 years post-diagnosis (Siegel et al., 2024). While surgery remains the primary treatment for PAAD, it is often accompanied by complications and trauma. Chemotherapy and radiotherapy are alternatives, yet despite progress, they have not substantially improved patient survival (Mizrahi et al., 2020).

Recent advances in systemic treatments, including novel chemotherapeutic agents and targeted therapies, have shown promise in improving outcomes for pancreatic cancer patients. For instance, emerging treatments have provided new insights into the therapeutic landscape and offer potential to extend patient survival (Sahin et al., 2024). Notably, targeted therapy, proven effective in numerous cancer types (Bhave et al., 2021; Chan et al., 2021; Garcia Campelo et al., 2021), has not been extensively explored in PAAD. Given that targeted therapy relies on cancer-specific target molecules, there is an urgent need to identify novel candidate targets in PAAD. In this context, recent efforts to directly inhibit oncogenic KRAS—one of the most prevalent driver mutations in PAAD—have gained increasing attention. Several KRAS^G12C^ inhibitors, such as sotorasib, have demonstrated preliminary efficacy and manageable safety in early clinical studies of pancreatic cancer patients (Hallin et al., 2020; Canon et al., 2019; Hong et al., 2020). Moreover, broader KRAS-targeted strategies, including KRAS^G12D^ inhibitors and pan-KRAS approaches, are actively being developed (Moore et al., 2020). Although KRAS^G12C^ mutations are rare (<2%) in PAAD, these advances highlight the feasibility of targeting RAS signaling in subsets of patients. This evolving therapeutic landscape underscores the pressing need to explore additional, potentially complementary, molecular targets in PAAD, such as *BMP4*.

Bone Morphogenetic Protein 4 (*BMP4*) plays a crucial role in mammalian development, particularly in tissues and organs originating from mesoderm and endoderm. As a ligand protein in the transforming growth factor-β superfamily, BMP4 typically binds to two receptors, BMPR1 and BMPR2, activating the Smad-dependent BMP signaling pathway. This activation leads to the phosphorylation of R-Smads (SMAD1, SMAD5, and SMAD8), forming a complex with common-partner Smads and initiating downstream gene transcription (Miyazono et al., 2010). Additionally, *BMP4* can regulate MAPK, PI3K/AKT, and Rho-GTPase pathways independently of Smads (Derynck and Zhang, 2003). Previous studies have reported upregulation of *BMP4* in various cancer types, including melanoma, gastric, and ovarian cancers (Rothhammer et al., 2005; Kim et al., 2011; Laatio et al., 2011). Dysregulation of *BMP4* is closely associated with cancer cell growth, apoptosis, migration, and invasion (Johnson et al., 2009; Hjertner et al., 2001; Virtanen et al., 2011). While some studies have investigated *BMP4*’s roles in PAAD (Virtanen et al., 2011; Hua et al., 2006; Gordon et al., 2009), they often utilized *in vitro* cultured cell lines and ectopically introduced *BMP4*, failing to delineate the roles of endogenously expressed *BMP4* orthotopically. Furthermore, the prognostic significance of *BMP4* in pancreatic cancer remains unclear.

In this study, we assessed *BMP4* expression in pan-cancer tissues and their matched normal counterparts, with notable dysregulation in PAAD. Bulk transcriptomic analyses identified differentially expressed genes associated with *BMP4*, and pathway analysis revealed that *BMP4* did not strongly affect canonical BMP signaling but instead significantly altered metabolic pathways. Single-cell transcriptomic data further showed *BMP4* expression in cancer cells and cancer stem cells. Finally, we demonstrated the prognostic significance of *BMP4* in PAAD. Together, these findings provide new insight into *BMP4* expression and function and underscore its potential role in prognosis prediction in PAAD.

## Materials and methods

### Pan-cancer analysis of *BMP4* expression and methylation

Relative levels of *BMP4* mRNA and protein in various normal tissues were obtained from the Genotype-Tissue Expression (GTEx) and Human Protein Atlas (HPA) databases. *BMP4* mRNA levels in pan-cancer cell lines were retrieved from the Cancer Cell Line Encyclopedia (CCLE) database. *BMP4* mRNA levels in cell lines from various normal tissues were downloaded from the HPA database. Clinical information, mRNA, and methylation profiles of patients across all cancer types were acquired from The Cancer Genome Atlas (TCGA). Expression and methylation data were visualized using the “ggplot2” package in R. Prognostic values of *BMP4* were assessed by dividing patients into high- and low-*BMP4* groups based on median levels, and survival analysis was conducted using the “survival” and “survminer” packages in R. Significance was set at P < 0.05, and results were visualized using the “ggplot2” package in R.

### RNA-sequencing (RNA-seq) data analysis

Transcriptome data of the PAAD cohort in TCGA (TCGA-PAAD) were analyzed. The GSE57495 and GSE78229 datasets were downloaded from the GEO database and served as validation cohorts. The clinical information of the TCGA-PAAD cohort is listed in Supplementary Table S1. Differentially expressed genes (DEGs) were determined using the “DESeq2” package in R with P < 0.05 and |log2(FoldChange)| > 1 as cutoffs. Gene ontology (GO) analysis was conducted using the “clusterProfiler” package in R and visualized using the “GOplot” package in R. Kyoto Encyclopedia of Genes and Genomes (KEGG) pathway analysis was performed at The Database for Annotation, Visualization and Integrated Discovery website (DAVID, https://david.ncifcrf.gov/). The results of DEG determination and KEGG analysis were visualized using the “ggplot2” package in R. P < 0.05 was used for the significance threshold. Protein-protein interaction (PPI) analysis was performed at the STRING website (https://cn.string-db.org/) and visualized using the Cytoscape software.

### Single-cell RNA sequencing (scRNA-seq) data analysis

scRNA-seq data of PAAD from GEO database (GSE197177, GSE214295, and GSE217845) were downloaded. GSE197177 was used as the test cohort, while GSE214295 and GSE217845 were used as the validation cohorts. Data were merged using the “harmony” package in R. The integrated scRNA-seq data was filtered using the following criteria: each cell should express 200–8,000 genes; each gene should be expressed in at least 3 cells; the ratio of mitochondrial genes should be less than 20%; the ratio of ribosomal genes should be more than 10%. Subsequently, the scRNA-seq data were subjected to doublet removal using the “DoubletFinder” package in R. Collectively, we obtained the filtered scRNA-seq data, including 24,495 genes and 22,639 cells. The scRNA-seq was primarily analyzed with the “Seurat” package in R. The Top 2000 genes were used for principal components analysis (PCA) and the top 40 principle components were used for Uniform Manifold Approximation and Projection (UMAP). By setting the resolution at 0.8, all cells in the scRNA-seq data were clustered into 23 clusters (clusters 0–22). The clusters were annotated with previously reported markers using the following criteria: B cells were *CD79A* and *CD79B* positive; cancer cells were *IMP3*, *MUC1*, and *S100P* positive and *PROM1* negative; cancer stem cells (CSCs) were *PROM1* positive; endocrine cells were *CHGA* and *CHGB* positive; endothelial cells were positive for *PECAM1*, *CDH5*, and *ENG*; epithelial cells were *CDH1*, *EPCAM*, and *KRTG19* positive and *IMP3*, *MUC1*, and *S100P* negative; fibroblasts were *ACTA2*, *COL11A1*, *COL1A1*, and *THY1* positive; macrophages were *AIF1*, *CD68*, and *CD86* positive; mast cells were *MS4A2* and *TPSAB1* positive; plasma cells were *CD27*, *CD38*, and *TNFRSF17* positive; T cells were *CD3D*, *CD3E*, and *CD3G* positive. DEGs were determined using the “FindMarkers” function in the Seurat package. All results were visualized using the built-in functions of the Seurat package and the “ggplot2” package in R.

### Kaplan-Meier survival analysis

The overall survival (OS), disease-specific survival (DSS), disease-free survival (DFS), and progression-free survival (PFS) of the TCGA-PAAD cohort were retrieved along with transcriptomic data. OS and transcriptomic data of validation cohorts (GSE57495 and GSE78229) were also downloaded. Kaplan-Meier survival analysis was performed using the “survival” and “survminer” packages in R. Patients were equally divided into two groups according to median levels of specific gene expressions or immune cell infiltrations. Significance was set at P < 0.05.

### Univariant and multivariant cox analyses

Univariant and multivariant Cox analyses were performed on the transcriptomic data and clinical information of the TCGA-PAAD cohort. Both Cox analyses were conducted using the “coxph” function in the “survival” R package with the gender, histological stages, age, and *BMP4* levels as the parameters. Univariant Cox analysis was performed with one of these parameters at a time, while multivariant Cox analysis used all these parameters together. The values of the hazard ratio, confidence intervals of 5% and 95%, and P-value were obtained from the Cox analyses and visualized using the “forestplot” package in R. The GSE57495 and GSE78229 datasets served as validation cohorts. P < 0.05 was considered to be significant.

### Nomogram analysis

Nomogram analysis was performed on the transcriptomic data and clinical information of the TCGA-PAAD cohort using the “rms” package in R. The predictive model of pancreatic cancer prognosis was constructed using gender, TNM stages, age, and *BMP4* levels as the parameters. Actual OS data were used for model verification. The GSE57495 and GSE78229 datasets served as validation cohorts.

### Immune infiltration analysis

Immune infiltration was determined using the CIBERSORT algorithm (v1.02) as described previously (Newman et al., 2015). The LM22 signature matrix was applied. The immune infiltration result included the relative infiltration levels of B cells (naïve, plasma, and memory), T cells (*CD8* ^+^, naïve *CD4* ^+^, resting memory *CD4* ^+^, activated memory *CD4* ^+^, follicular helper, regulatory, and gamma delta), NK cells (resting and activated), macrophages (M0, M1, and M2), dendritic cells (resting and activated), mast cells (resting and activated), monocytes, eosinophils, neutrophils. All results were visualized using the “ggplot2” package in R. P < 0.05 was considered to be significant.

### Immunohistochemical staining

Immunohistochemical staining was conducted using an Immunohistochemistry Kit (Sangon Biotech, Cat# D601037) according to the manufacturer’s protocol. Pancreatic cancer and its para-carcinoma slides were first dewaxed with xylene and rehydrated with a gradient concentration of ethanol. The slides were then sequentially subjected to antigen retrieval, bovine serum albumin (BSA) blocking, and overnight incubation of primary antibodies. On the next day, the slides were incubated with HRP-conjugated secondary antibodies and then subjected to a chromogenic reaction. The slides were further stained with hematoxylin. Images were captured using a Leica DMi8 fluorescence microscope. The antibodies used in this study were as follows: BMP4 mAb (Abclonal, Cat# A11405) and Goat Anti-Rabbit HRP secondary antibody (Biosharp, Cat# BL003A). All clinical samples used in this study were obtained with written informed consent from patients. Our study and all methods were approved by the Ethics Committee of Anhui Normal University.

### Cell culture

PANC-1 cells, which exhibited low basal *BMP4* expression, were cultured in the Dulbecco’s Modified Eagle’s Medium (DMEM, Gibco) supplemented with 10% Fetal Bovine Serum (FBS), 50 U/mL penicillin (Gibco), and 50 μg/mL streptomycin (Gibco). For BMP4 treatment, 10 ng/mL BMP4 (R&D systems) was added.

### Real-time quantitative PCR (RT-qPCR)

Total RNA was extracted with a total RNA isolation reagent (Biosharp). Reverse transcription was performed with the FastKing RT kit (Tiangen) and quantitative PCR was performed with the Powerup SYBR master mix (Applied Biosystems). These experiments were conducted according to their corresponding manufacturer’s protocols. *β-ACTIN* was used as the internal control. All primer sequences used in this study are listed in Supplementary Table S2.

## Results

### Pan-cancer analysis of *BMP4* expressions and prognostic values

BMP4 is an important ligand regulating development and diseases via the BMP signaling pathway, while it is also involved in the regulation of other signaling pathways. Previous studies have demonstrated the essential roles of *BMP4* and the BMP signaling pathway in the development and certain cancer types. However, a systematic study on the profile and prognostic values of *BMP4* from a pan-cancer perspective was still lacking. Here, we first explored the expression patterns of *BMP4* in normal tissues. We analyzed the transcriptome and proteome data from the HPA and GTEx databases. Both the RNA and protein of *BMP4* were highly expressed in the prostate, ovary, colon, etc., while lowly expressed in the pancreas, substantia nigra, putamen, liver, etc. (Supplementary Figure S1A). In contrast, by analyzing the relative expressions of *BMP4* in various cell lines of different cancer types, we found that *BMP4* was highly expressed in the cancers of the pancreas, eye, liver, etc. (Supplementary Figure S1B). It should be noted that *BMP4* was relatively low expressed in pancreas but aberrantly upregulated in pancreatic cancer, implying a potential involvement of *BMP4* in the tumorigenesis of pancreatic cancer. Furthermore, we determined the detailed distributions of *BMP4* in various cell types of normal tissues. For most tissues, *BMP4* was enriched in fibroblasts, smooth muscle cells, and endothelial cells (Supplementary Figure S1C). This agreed with previously documented BMP4-secreting cell types. We next investigated the prognostic values of *BMP4* in all cancer types listed in the TCGA database, the outcomes of which were highly divergent according to different cancer types. When focusing on the overall survival (OS), *BMP4* was a significant hazard factor for adrenocortical cancer (ACC), pancreatic cancer (PAAD), and pheochromocytoma & paraganglioma (PCPG) and a beneficial factor for breast cancer (BRCA), acute myeloid leukemia (LAML), lower grade glioma (LGG), and stomach cancer (STAD). Particularly, in addition to OS, *BMP4* was also a beneficial factor for the disease-specific survival (DSS), disease-free survival (DFS), and progression-free survival (PFS) of STAD and a hazard factor for the DFS and PFS of PAAD (Supplementary Figure S1D). Taken together, the contrast expression patterns of *BMP4* in normal pancreas and pancreatic cancer and its prognostic values implied an important role of *BMP4* in PAAD.

### Pan-cancer analysis of *BMP4* methylations

DNA methylation is an important epigenetic regulation of gene expression and its dysregulation serves as a hallmark of tumorigenesis. Hypermethylation was usually associated with suppressed gene expressions, while hypomethylation usually led to enhanced gene expressions. As we have shown that the expressions of *BMP4* were largely dysregulated during tumorigenesis, we wondered if such expression changes were due to DNA methylation. We determined the relative methylation levels of *BMP4* locus in various cancer types and found that its methylation was relatively low in testicular cancer (TGCT), PCPG, and mesothelioma (MESO) but relatively high in thyroid cancer (THCA), PAAD, and lung adenocarcinoma (LUAD) (Supplementary Figure S2A). Next, we analyzed the correlation between the expression and methylation of *BMP4* in all cancer types. As expected, the methylation and expression of *BMP4* were adversely correlated in most cancer types, with such correlations being significant only in endometrioid cancer (UCEC), lung squamous cell carcinoma (LUSC), LAML, thymoma (THYM), ocular melanomas (UVM), ACC, uterine carcinosarcoma (UCS), and large B-cell lymphoma (DLBC) (Supplementary Figure S2B). Subsequently, we explored the prognostic values of *BMP4* methylation in all cancer types. *BMP4* methylation was a significantly beneficial factor for the OS of ACC, glioblastoma (GBM), LGG, prostate cancer (PRAD), and UCEC. Besides OS, *BMP4* methylation was also beneficial to the DSS of ACC, DSS, and PFS of GBM and LGG, PFS of PAAD, DSS, DFS, and PFS of UCEC. In contrast, *BMP4* methylation was hazardous for the DSS of MESO and PCPG, PFS of PRAD, and DFS of sarcoma (SARC) (Supplementary Figure S2C). It is worth noting that *BMP4* expression was significantly hazardous for the OS of PAAD, while its methylation did not affect the OS of PAAD. This agreed with the correlation of expression and methylation of *BMP4* in PAAD, which was not significant. These results indicated that the methylation of *BMP4* was not the main cause of its aberrant upregulation and had little effect on prognosis in PAAD.

### *BMP4* was significantly upregulated and associated with poor prognosis in PAAD

**FIGURE 1:**
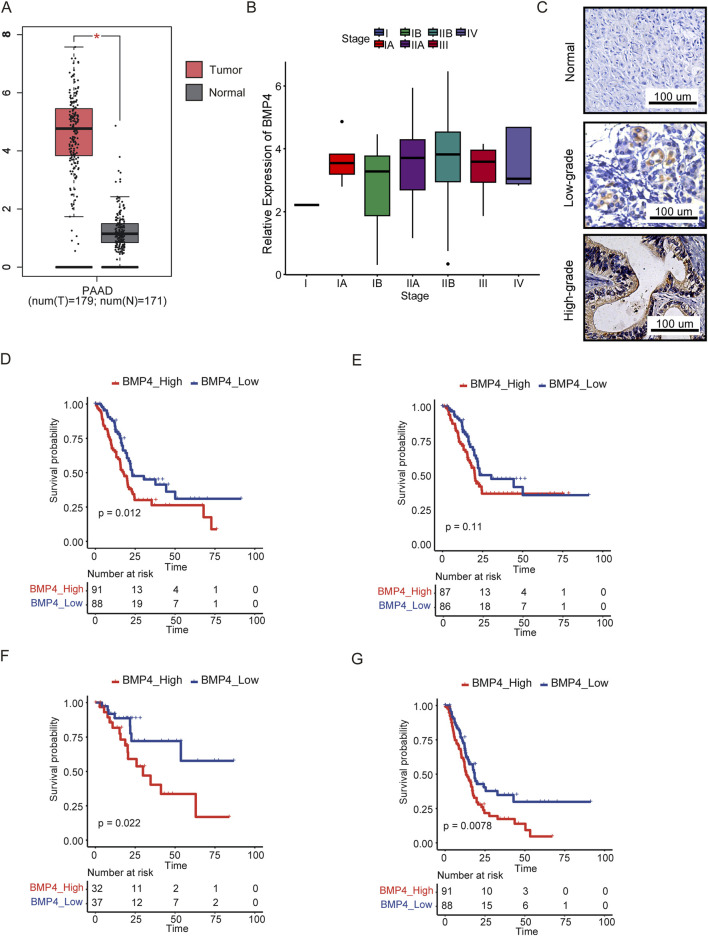
*BMP4* was upregulated and associated with poor prognosis in the TCGA-PAAD cohort. **(A)** The relative levels of *BMP4* in PAAD and normal pancreas tissues. **(B)** The relative levels of *BMP4* in different stages of PAAD. **(C)** Immunohistochemistry against BMP4 in low-grade PAAD, high-grade PAAD, and para-carcinoma patient samples. The Kaplan-Meier survival curves compared the **(D)** OS, **(E)** DSS, **(F)** DFS, and **(G)** PFS between the patients with high- and low-*BMP4* expressions. *, P < 0.05.

As we have shown that *BMP4* was initially low expressed in normal pancreas tissues and became the top expressed in PAAD among all cancer types, we determined to systematically study the function and prognostic values of *BMP4* in PAAD. We merged the transcriptomic data of the PAAD cohort in the TCGA with that of the normal pancreas in the GTEx. Compared to the normal pancreas, *BMP4* was significantly upregulated in the PAAD (Figure 1A). The expression of *BMP4* showed an escalating trend as the pathological stage progressing (Figure 1B) but was not significantly affected by the age, gender, and recurrent status of PAAD patients (Supplementary Figure S3A-C). For verification, we performed immunohistochemistry against BMP4 on normal pancreas, low-grade PAAD, and high-grade PAAD tissues. As a result, *BMP4* was remarkably expressed in both low-grade and high-grade PAAD tissues but not in the normal pancreas, with the high-grade PAAD showing stronger *BMP4* expressions (Figure 1C). We next evaluated the impacts of *BMP4* on the OS, DFS, DSS, and PFS of PAAD using the Kaplan-Meier survival curves, respectively. All survival assays were conducted by clustering patients into the high- and low-*BMP4* groups according to the median expression of *BMP4*. As a result, the OS, DFS, and PFS of PAAD were significantly shorter with high *BMP4* expressions, while the DSS was unaffected (Figures 1D–G).

**FIGURE 2:**
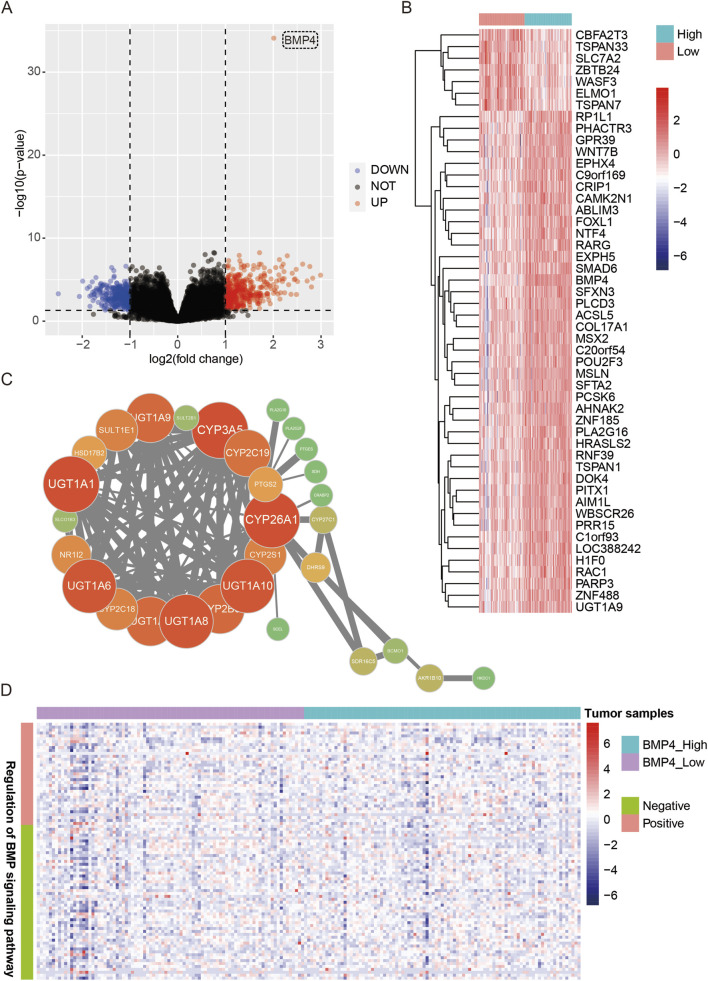
The gene expression differences between the TCGA-PAAD patients with high-and low-*BMP4* expressions. **(A)** The volcano plot showing the DEGs between the PAAD patients with high- or low-*BMP4* expressions. **(B)** The heatmap showing the relative levels of the top 50 level-changed DEGs. **(C)** The protein-protein interactions between the upregulated DEGs. **(D)** The heatmap showing the relative levels of the genes regulating the BMP signaling pathway.

Given that *BMP4* upregulation was significantly hazardous for the prognosis of PAAD, we are curious about the gene expression changes caused by *BMP4* dysregulation. We equally divided the patients in the PAAD cohort of the TCGA database into the low- and high-*BMP4* groups according to the median expression of *BMP4*. Subsequently, we determined the differentially expressed genes (DEGs) between the two groups using the “DESeq2” package in R. P-value <0.05 and |log2(Fold Change)|>1 were used as a significance threshold. Collectively, there were 419 genes upregulated and 305 genes downregulated with *BMP4* upregulation (Figure 2A). We also plotted a heatmap of the top 50 altered genes and built a protein-protein interaction network of essential node genes. By looking into the DEGs, we noticed that *WNT7B*, *SMAD6*, *TSPAN1*, and *PARP3* were among the top altered DEGs regarding *BMP4* upregulation (Figures 2B,C). Such genes were related to the Wnt and TGF-β signaling pathways and the DNA repair process, which has been implicated as important for regulating pancreatic cancer (Ram Makena et al., 2019; Zhao et al., 2018; Perkhofer et al., 2021).

**FIGURE 3:**
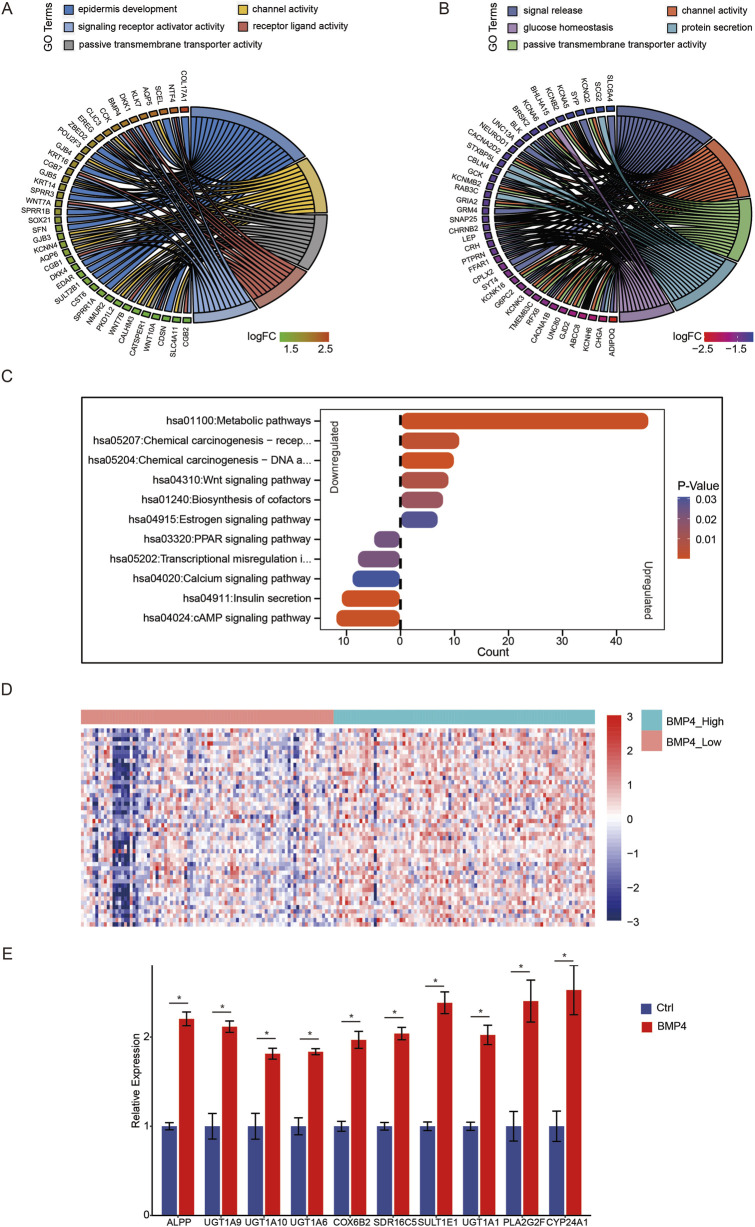
The changes in biological processes and pathways due to *BMP4* alterations in the TCGA-PAAD cohort. The GO terms of **(A)** upregulated and **(B)** downregulated DEGs. **(C)** The pathways that were significantly altered with *BMP4* dysregulation. **(D)** The heatmap showing the relative levels of the genes associated with metabolic pathways. **(E)** RT-qPCR against the top 10 altered metabolism-related DEGs in BMP4 treated and untreated PANC-1 cells. *, P < 0.05.

As BMP4 was a well-known ligand for the BMP signaling pathway, we asked if the upregulation of *BMP4* indeed led to the activation of the BMP signaling pathway in PAAD. We extracted the expression levels of the genes related to the gene ontology (GO) terms of both positive and negative regulation of the BMP signaling pathway. We found that those BMP signaling pathway-related genes were all unaffected in response to *BMP4* alterations, indicating that *BMP4* levels might not consequently alter the BMP signaling pathway in PAAD (Figure 2D). As such, we performed gene ontology (GO) and KEGG pathway analyses to reveal the biological processes and pathways that *BMP4* regulated in PAAD. As a result, we found that *BMP4* promoted epidermis development, channel activity, signaling receptor activator activity, receptor-ligand activity, and passive transmembrane transporter activity-related processes (Figure 3A). However, *BMP4* suppressed the processes of signal release, channel activity, glucose homeostasis, protein secretion, and passive transmembrane transporter activity (Figure 3B). As to pathways, *BMP4* mainly promoted the metabolic, chemical carcinogenesis, Wnt signaling, biosynthesis of cofactors, and estrogen signaling-related pathways, but suppressed the PPAR signaling, calcium signaling, insulin secretion, and cAMP signaling pathways. Notably, there were 46 DEGs prominently associated with the metabolic pathways, which was top-ranked as to total gene counts enriched in each pathway, suggesting *BMP4* might facilitate tumorigenesis via adjusting metabolism (Figure 3C). We plotted a heatmap showing the relative expressions of the 46 metabolism-related DEGs in the PAAD samples of the TCGA and found that these genes were relatively highly expressed in the high-*BMP4* group (Figure 3D). Moreover, consistent with the above findings, the BMP signaling was not among the significantly changed pathways. The *BMP4*-associated metabolic genes were mainly enriched in pathways related to drug/xenobiotic metabolism (*UGT1A* family), lipid and phospholipid metabolism (*PLA2G2F*, *SDR16C5*), steroid/estrogen metabolism (*SULT1E1*, *UGT1A1*), vitamin D and A metabolism (*CYP24A1*, *SDR16C5*), and mitochondrial oxidative phosphorylation (*COX6B2*). These results suggested that *BMP4* might broadly reprogram pancreatic cancer cell metabolism at multiple levels. For validation, we re-analyzed the significantly altered pathways in response to *BMP4* expression changes in two validation cohorts, GSE57495 and GSE78229. Similarly, metabolic pathways were the most significantly promoted pathways in both cohorts (Supplementary Figure S4A,B). The DEGs related to metabolism in both cohorts were relatively highly expressed with high *BMP4* expressions (Supplementary Figure S4C,D). To further validate the effect of *BMP4* on the metabolism of PAAD, we compared the expressions of the top ten altered metabolism-related DEGs between the BMP4-treated and -untreated PANC-1 cells, of which the endogenous *BMP4* was relatively low expressed among PAAD cell lines (Supplementary Figure S5). Consistently, these DEGs were also significantly upregulated with *BMP4* treatment (Figure 3E).

### *BMP4* was mainly expressed by cancer cells and cancer stem cells

**FIGURE 4:**
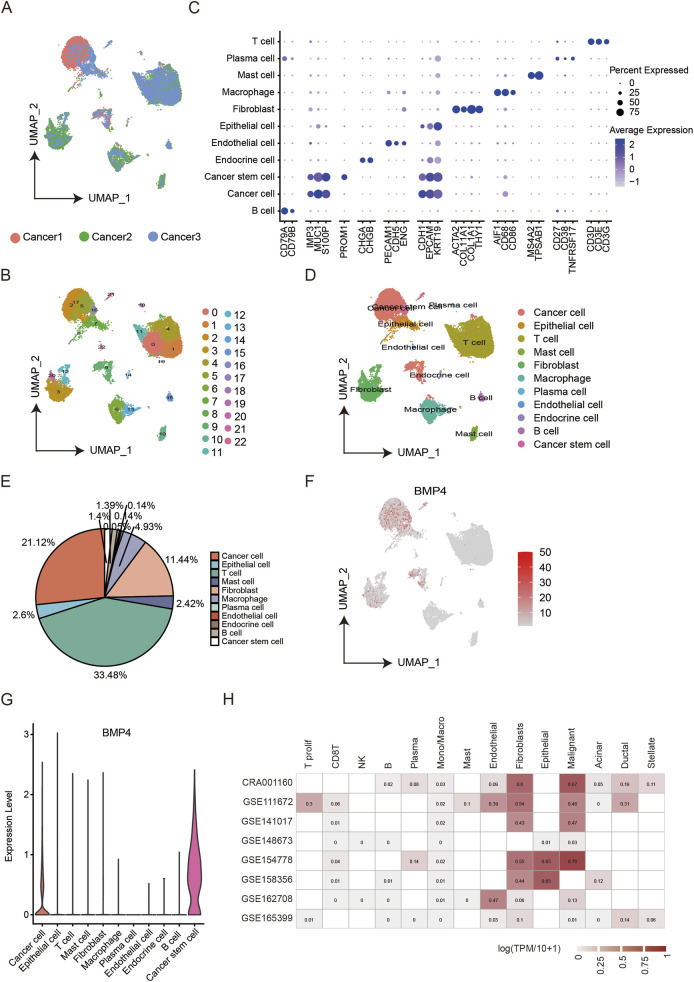
The analysis of the PAAD scRNA-seq dataset GSE197177. **(A)** The UMAP plot showing the cells from three patients. **(B)** The UMAP plot showing 23 clusters within the PAAD scRNA-seq data. **(C)** The relative levels of cell markers used for annotation in different cell subgroups. **(D)** The UMAP plot showing different cell subgroups. **(E)** The ratios of different cell subgroups in the scRNA-seq data. **(F)** The UMAP plot showing BMP distributions in the scRNA-seq data. **(G)** The relative levels of *BMP4* in each cell subgroup. **(H)** The relative levels of *BMP4* in different cell types of various PAAD cohorts.

Single-cell RNA sequencing (scRNA-seq) has been emerging as an approach to resolve cell constitutions within specific tissues or cell culture samples. Given the significant upregulations during tumorigenesis and prognostic values of *BMP4* in PAAD, we would like to determine the cell types within PAAD tumors where *BMP4* was expressed. We analyzed scRNA-seq data of PAAD, which consisted of cells collected from three PAAD patients. The scRNA-seq data were subjected to data filtration and dimension reduction. By UMAP projection, the cells from the three patients displayed similar cell distributions, suggesting that the cell compositions identified here were generally representative (Figure 4A). Next, we used the built-in function, “FindClusters”, in the “Seurat” package and set the resolution at 0.8 to discover clusters within the scRNA-seq data. Consequently, we obtained 23 clusters (clusters 0–22) (Figure 4B). We then annotated these cell clusters using previously reported cell markers (Figure 4C). Collectively, the scRNA-seq data contained 11 cell types (Figure 4D) and the top three enriched cell types were T cells (33.48%), cancer cells (21.12%), and fibroblasts (11.44%) (Figure 4E). Subsequently, we investigated the distributions of *BMP4* among these cell types. As a result, we found that *BMP4* was specifically enriched in CSCs and cancer cells, and was also clearly expressed in epithelial cells and fibroblasts (Figures 4F,G). Consistent with this, we retrieved the transcriptomic profile data of different cohorts of PAAD and found that the expressions of *BMP4* were enriched in the malignant cancer cells, epithelial cells, and fibroblasts (Figure 4H). For validation, we re-performed the same analyses on the GSE214295 and GSE217845 cohorts. With the same cell-specific markers, we successfully annotated all the above cell types. Consistently, *BMP4* was also discovered to be expressed in CSCs and cancer cells (Supplementary Figures S6,S7). These results together showed that *BMP4* was primarily expressed in cancer cells and CSCs. However, as BMP4 is a secreting protein, its protein distribution might differ from its transcriptomic pattern, as indicated by immunohistochemistry results. Nevertheless, the intrinsic mechanism that controlled the specific transcriptomic expressions of *BMP4* warrants further investigation.

**FIGURE 5:**
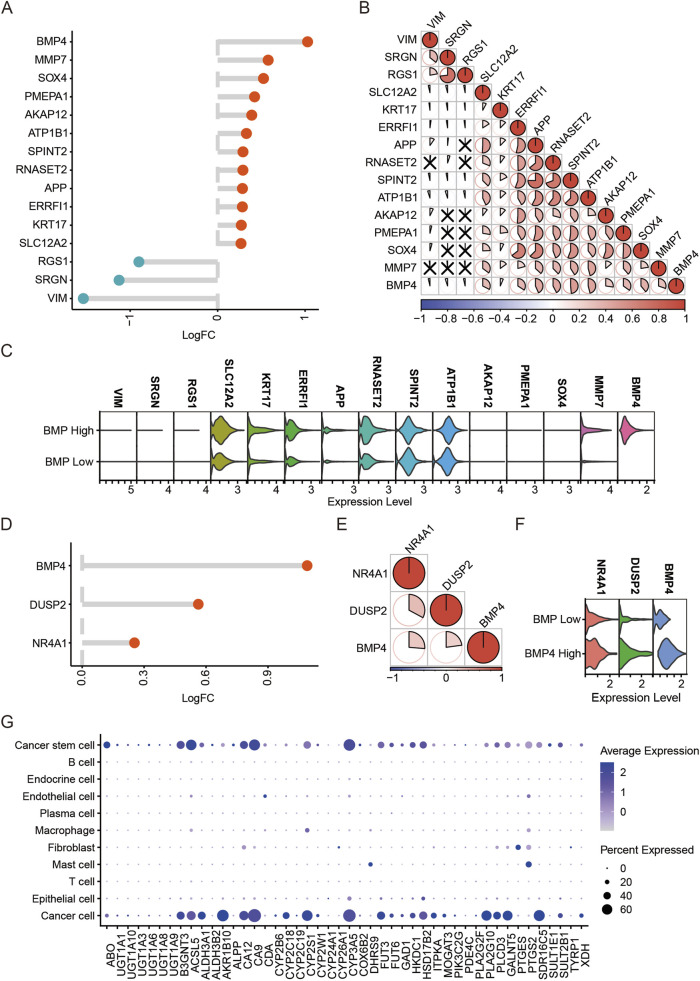
The DEGs between the high-and low-*BMP4* cancer cells and CSCs in the PAAD scRNA-seq dataset GSE197177. **(A)** The log2|(Fold Change)| of the DEGs with *BMP4* level changes in the cancer cells of PAAD. **(B)** The correlations between the DEGs in **(A)**. **(C)** The relative levels of top DEGs in the high- and low-*BMP4* cancer cells. **(D)** The log2|(Fold Change)| of the DEGs with *BMP4* level changes in the CSCs of PAAD. **(E)** The correlations between the DEGs in **(D)**. **(F)** The relative levels of top DEGs in the high- and low-*BMP4* CSCs. **(G)** The relative levels of metabolic pathway-associated genes in different cell subgroups of the PAAD scRNA-seq data.

As *BMP4* was enriched in the cancer cells and CSCs, we extracted these two types of cells from the scRNA-seq data and studied the impacts of *BMP4* level changes on the expressions of other genes, respectively. First, we extracted the cancer cell subgroup and determined the DEGs associated with *BMP4* dysregulations. By equally dividing the cancer cells into two groups, high- and low-*BMP4* cells, according to cell-intrinsic *BMP4* expressions, we analyzed the DEGs using a built-in function, “FindMarkers”, in the “Seurat” package. By setting P-value <0.05 and |logFC| > 0.25 as the therashold, we found that *MMP7*, *SOX4*, *PMEPA1*, *AKAP12*, *ATP1B1*, *SPINT2*, *RNASET2*, *APP*, *ERRFl1*, *KRT17*, and *SLC12A2* were significantly upregulated, while *RGS1*, *SRGN*, and *VIM* were significantly downregulated (Figures 5A-C). We analyzed the correlations between these DEGs with *BMP4* and found that *SOX4*, *ATP1B1*, *SPINT2*, *RNASET2*, and *APP* were among the top correlated ones (Figure 5B). Subsequently, we conducted similar analyses on the CSCs. Using the same criteria for DEG determination, we only identified *DUSP2* and *NR4A1* as the significantly upregulated genes in the high-*BMP4* CSCs when compared to the low-*BMP4* group (Figures 5D-F). By comparison, *DUSP2* was more closely correlated with *BMP4* (Figure 5E). Similarly, BMP4 treatment on PANC-1 cells significantly triggered the upregulation of *DUSP2* (Supplementary Figure S8). Emerging studies have suggested that *DUSP2* served as a favorable factor for cancer metastasis, chemoresistance, and cancer stemness (Hou et al., 2017; Zhang et al., 2022). The close association between *DUSP2* and *BMP4* in the CSCs attracts further efforts to depict the role of *BMP4* in the CSCs of PAAD. As the KEGG pathway analysis on the TCGA-PAAD cohort suggested that *BMP4* might manipulate metabolism pathways, we studied the relative levels of the metabolism-relative DEGs in all cell types of the scRNA-seq data. Similar to *BMP4*, these DEGs were also enriched in the cancer cells and CSCs (Figure 5G).

### *BMP4* could be applied into the prediction of PAAD prognosis

**FIGURE 6:**
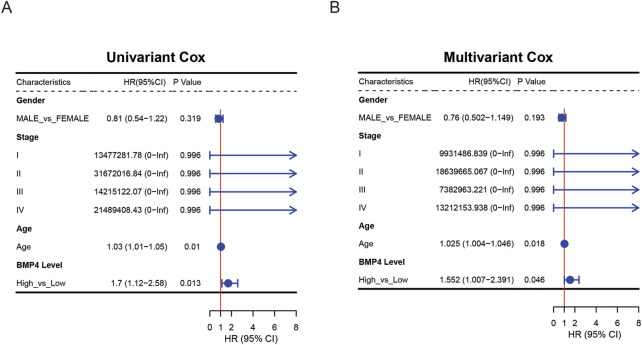
The expression of *BMP4* was an independent prognostic factor for PAAD. **(A)** Univariant and **(B)** multivariant Cox regression analyses were performed on *BMP4* expressions and other clinical parameters. These analyses were all performed on the TCGA-PAAD cohort.

**FIGURE 7:**
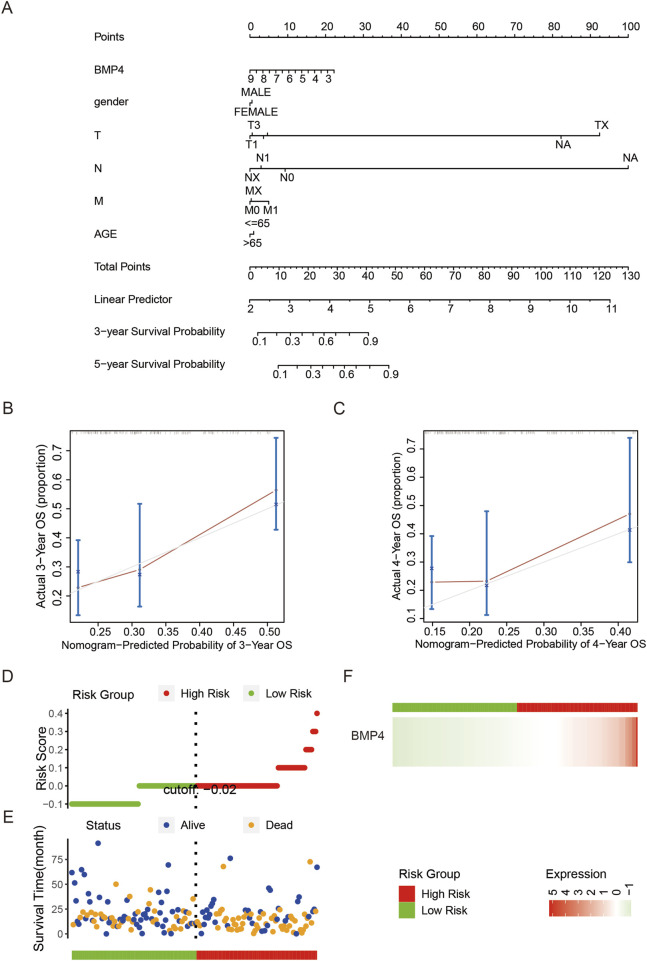
*BMP4* could be used for prognosis prediction for PAAD. **(A)** A nomogram prognosis model was constructed with *BMP4* expressions and other clinical parameters. The validation of the nomogram model using the actual **(B)** 3- and **(C)** 4–years OS data. **(D)** The patients equally divided into high- and low-risk groups. **(E)** The vital status and survival intervals of the PAAD patients in the high- and low-risk groups. **(F)** The relative expressions of *BMP4* in the high- and low-risk groups. These analyses were all performed on the TCGA-PAAD cohort.

To study whether *BMP4* is an independent prognostic factor for PAAD, we performed sequential univariant and multivariant Cox analyses on the PAAD cohort of the TCGA database. The parameters included in the Cox analyses were gender, histological stages, age, and BMP levels. The univariant and multivariant Cox analyses suggested that both *BMP4* levels and age were independent hazard factors for the PAAD prognosis, while gender and histological stages were not (Figure 6). For validation, we performed the univariant and multivariant Cox analyses on the validation cohorts, GSE57495 and GSE78229. Since the two validation cohorts did not provide age and gender information, the parameters included in the Cox analyses performed in the validation cohorts only contained histological stages and *BMP4* expressions. Consequently, *BMP4* was an independent hazard factor in both cohorts (Supplementary Figure S9). Thus, we tested if *BMP4* levels could be applied into the prediction of PAAD prognosis along with other parameters. For this purpose, we attempted to establish a nomogram model consisting of *BMP4* expressions, gender, TNM stages, and age. Consistently, the constructed nomogram model suggested that high *BMP4* levels were associated with low survival probabilities (Figure 7A). To validate this model, we compared the predicted survival probabilities from this model to the actual clinical survival data and found that the predicted survival probabilities well obeyed the actual data, implying its potential application in the prognosis prediction of PAAD (Figures 7B,C).

We also calculated the risk scores for all patients in the TCGA cohort using the “ggrisk” R package. By equally dividing all patients into high- and low-risk groups according to their risk scores, we found that the patients in the high-risk group were of relatively shorter survival time and higher mortality (Figures 7D,E). Notably, *BMP4* was specifically highly expressed in the high-risk group, orchestrating its hazard role in PAAD prognosis (Figure 7F). As the above studies were all performed on the PAAD cohort of TCGA, we here employed two validation cohorts, GSE57495 and GSE78229, to validate the prognostic value of *BMP4* and thus exclude the possibility that the effect of *BMP4* on PAAD was only restricted to TCGA cohort. In line with our above findings, the patients with higher *BMP4* expression displayed significantly shorter OS in both validation cohorts (Supplementary Figure S10A,B). Thus, *BMP4*, a factor specifically enriched in the cancer cells and CSCs of PAAD, could serve as a potential prognostic marker for PAAD.

### *BMP4* affected PAAD prognosis not via regulating immune infiltrations

**FIGURE 8:**
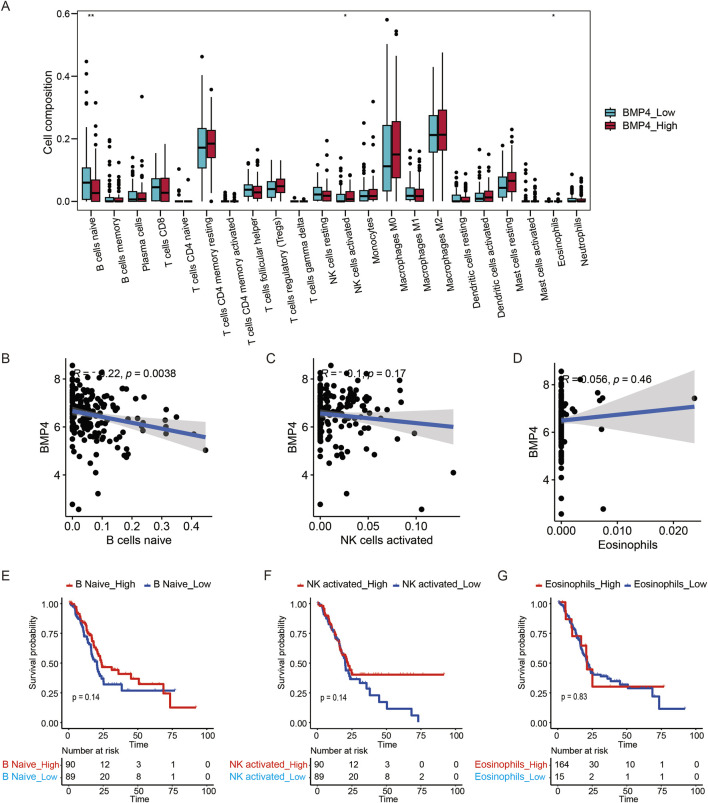
*BMP4* affected PAAD prognosis not via regulating immune infiltrations. **(A)** Certain immune cell infiltrations were affected by altered *BMP4* expressions. **(B–D)** The correlations between the *BMP4* levels and the significantly altered immune infiltrations. The Kaplan-Meier survival curves showing the OS of PAAD patients with different infiltration levels of **(E)** naïve B cells, **(F)** activated NK cells, and **(G)** eosinophils, respectively. *, P < 0.05; **, P < 0.01. These analyses were all performed on the TCGA-PAAD cohort.

As immune infiltrations within tumors have profound impacts on tumorigenesis, metastasis, and prognosis, we wondered whether *BMP4* affected PAAD prognosis through immune infiltrations. First, we employed the CIBERSORT algorithm to evaluate the overall infiltrations of 22 types of immune cells. The infiltrations of naïve B cells, activated NK cells, and Eosinophils were significantly affected by *BMP4* levels (Figure 8A). Next, we studied the correlations between *BMP4* expressions and these immune infiltrations. Only naïve B cells were significantly negatively correlated with *BMP4* levels (Figures 8B–D). Moreover, we investigated the impacts of these three immune infiltrations on PAAD prognosis and found that none of these infiltrations significantly affected PAAD prognosis (Figures 8E–G). These results together indicated that *BMP4* regulated PAAD prognosis not through manipulating immune infiltrations.

## Discussion

*BMP4* is essential in development, particularly for mesoderm and endoderm, but its role in tumorigenesis is less understood. Pancreatic cancer remains highly lethal with poor survival, and whether *BMP4* contributes to its progression was unclear. By integrating bulk and single-cell transcriptomic data, we revealed that *BMP4* is enriched in cancer cells and CSCs, correlates with poor prognosis, and shows a distinctive role in regulating metabolism rather than canonical BMP signaling. A nomogram incorporating *BMP4* further demonstrated its prognostic value.

*BMP4*, as a member of the TGF-β superfamily, orchestrates embryonic development and organogenesis (Massague, 2000). Its essential roles in the heart, eye, limb, and various tissues have been elucidated through expression patterns and functional analyses. (Goldman et al., 2009; Li et al., 2008; Domyan et al., 2011; Pizette and Niswander, 2000). Canonically, BMP4 activates SMAD1/5/8 through BMPR1/2 receptors, while non-canonical signaling involves MAPK, PI3K/AKT, and Rho-GTPases (Derynck and Zhang, 2003; Gipson et al., 2020). *BMP4* also cross-talks with other pathways such as Wnt/β-catenin (Bottasso-Arias et al., 2022). In our study, however, *BMP4* did not primarily activate BMP signaling but promoted metabolic pathways, as confirmed by the upregulation of metabolism-related genes in both bulk and single-cell data. These findings suggested that *BMP4* might broadly reprogram tumor metabolism, influencing drug metabolism, lipid signaling, energy production, and hormone/vitamin pathways. Such multi-level metabolic regulation may represent a novel mechanism through which *BMP4* promotes PAAD progression. This aligns with prior reports linking *BMP4* to metabolic diseases, including obesity, diabetes, and hepatic steatosis (Son et al., 2011; Wang et al., 2017; Modica et al., 2016; Peng et al., 2019), suggesting that *BMP4* may facilitate PAAD tumorigenesis via metabolic regulation.

BMP4 is mainly produced by the stem cell niche and can act through paracrine, autocrine, or endocrine modes (Son et al., 2011; Qi et al., 2004; Jones and Wagers, 2008). We found *BMP4* to be low in normal pancreas but aberrantly upregulated in PAAD, particularly in cancer cells and CSCs, consistent with its origins in stem cell populations. Interestingly, *BMP4* was also upregulated in tumor vasculature, implying potential secretion, although proteomic datasets (the PXD009139 cohort deposited in the Proteomics Identifications Database (PRIDE) and the IPX0001579000 cohort deposited in the Integrated Proteome Resources database (iProX)) did not detect BMP4 in serum. This suggests BMP4 may remain localized, and more sensitive assays will be needed to test its value as a circulating biomarker.

Immune infiltration is a critical component of tumorigenesis. *BMP4* has been implicated in *CD4* ^+^ T cell activation and M2 macrophage polarization (Martinez et al., 2015; Martinez et al., 2017). In our study, *BMP4* expression correlated with altered infiltration of naïve B cells, activated NK cells, and eosinophils. However, these changes did not account for the prognostic impact of *BMP4*, suggesting its role is largely independent of immune modulation. Functional assays are warranted to clarify how *BMP4*-driven immune changes influence PAAD biology.

This study has several limitations. Most analyses relied on public datasets, and although validated across multiple cohorts, additional clinical data would strengthen our conclusions. The precise mechanisms by which *BMP4* regulates metabolism remain unclear and should be addressed in future mechanistic studies. The prognostic model requires further clinical validation. Finally, while *BMP4* influenced immune infiltration, its lack of prognostic relevance in this context also needs further investigation.

Despite these limitations, our findings highlight the multifaceted roles of *BMP4* in PAAD. By emphasizing its metabolic regulation, prognostic significance, and enrichment in cancer cells and CSCs, this study provides a foundation for future work exploring *BMP4* as both a biomarker and a therapeutic target in pancreatic cancer.

## Data Availability

The datasets presented in this study can be found in online repositories. The names of the repository/repositories and accession number(s) can be found in the article/Supplementary Material.
