# Supplementary material for: Comprehensive analyses of single-cell and bulk RNA-seq reveal the biological and prognostic roles of BMP4 in pancreatic adenocarcinoma
Source: Front Mol Biosci. 2025 Oct 15;12:1686938. doi: 10.3389/fmolb.2025.1686938 (PMC12568421; doi:10.3389/fmolb.2025.1686938)
Supplement: Supplementary file 1 [file DataSheet1.zip › supplmentary materials/Table S1.docx]

**Table S1** Clinical characteristics of the TCGA-PAAD cohort

|  | **BMP4_High (N=91)** | **BMP4_Low (N=88)** | **P-value** |
| --- | --- | --- | --- |
| **gender** |  |  |  |
| MALE | 56 (61.5%) | 43 (48.9%) | 0.12 |
| FEMALE | 35 (38.5%) | 45 (51.1%) |  |
| **T** |  |  |  |
| TX | 0 (0%) | 1 (1.1%) | 0.28 |
| T1 | 3 (3.3%) | 4 (4.5%) |  |
| T2 | 8 (8.8%) | 16 (18.2%) |  |
| T3 | 78 (85.7%) | 65 (73.9%) |  |
| T4 | 2 (2.2%) | 1 (1.1%) |  |
| NA | 0 (0%) | 1 (1.1%) |  |
| **N** |  |  |  |
| NX | 1 (1.1%) | 3 (3.4%) | 0.38 |
| N0 | 23 (25.3%) | 27 (30.7%) |  |
| N1 | 67 (73.6%) | 57 (64.8%) |  |
| NA | 0 (0%) | 1 (1.1%) |  |
| **M** |  |  |  |
| MX | 48 (52.7%) | 46 (52.3%) | 0.886 |
| M0 | 41 (45.1%) | 39 (44.3%) |  |
| M1 | 2 (2.2%) | 3 (3.4%) |  |
| **stage** |  |  |  |
| I | 7 (7.7%) | 14 (15.9%) | 0.228 |
| II | 80 (87.9%) | 67 (76.1%) |  |
| III | 2 (2.2%) | 2 (2.3%) |  |
| IV | 2 (2.2%) | 3 (3.4%) |  |
| NA | 0 (0%) | 2 (2.3%) |  |
| **AGE** |  |  |  |
| <=65 | 50 (54.9%) | 44 (50.0%) | 0.608 |
| >65 | 41 (45.1%) | 44 (50.0%) |  |
| **vital_status** |  |  |  |
| ALIVE | 35 (38.5%) | 51 (58.0%) | 0.0139 |
| DEAD | 56 (61.5%) | 37 (42.0%) |  |

TCGA, The Cancer Genome Atlas; LUAD, Pancreatic Adenocarcinoma; NA, not applicable.
