# Supplementary material for: Comprehensive analyses of single-cell and bulk RNA-seq reveal the biological and prognostic roles of BMP4 in pancreatic adenocarcinoma
Source: Front Mol Biosci. 2025 Oct 15;12:1686938. doi: 10.3389/fmolb.2025.1686938 (PMC12568421; doi:10.3389/fmolb.2025.1686938)
Supplement: Supplementary file 1 [file DataSheet1.zip › supplmentary materials/Table S2.docx]

**Table S2** List of primers used for RT-qPCR

| **Genes** | **Forward Primer** | **Reverse Primer** |
| --- | --- | --- |
| ALPP | ATCTTCCTGGGCGATGGGAT | CACATATGGGAAGCGGTCCA |
| UGT1A9 | CGGAGTATGATCTCTACAGCCAC | TACCGCCACAGGACTGTCT |
| UGT1A10 | TGGAGGACCATTTATTTTGCCAG | AGGCTTCAAATTCCATAGGCA |
| UGT1A6 | GACTTTTTCCCAACGAGTGGC | AGGCTTCAAATTCCTGAGACA |
| COX6B2 | CAAAGCCCTAGGGTTCCAGC | GCTCATCTAGGGTGTGTCCTTT |
| SDR16C5 | AGGTTGTACTACAGGCTGTCC | GCTCTGTCCCACATAGCCTC |
| SULT1E1 | TTCATGCAAGGACAGGTTCC | CCTGTCCACAAGCTCCTCTG |
| UGT1A1 | AACTGCCTTCACCAAAATCCAC | GTCATCGGGTGACCAAGCA |
| PLA2G2F | CTGAGATAGTCTGCAGTGACC | CGCTCTCTCTCTCAAACCTCAG |
| CYP24A1 | AGCGATAATACGCCTCAGATGG | TGCAAGACAGAACAGGCTCC |
| DUSP2 | TACTTCCTGCGAGGAGGCTT | CCAGGAACAGGTAGGGCAAG |
| ACTB | CACCATTGGCAATGAGCGGTTC | AGGTCTTTGCGGATGTCCACGT |
