# Supplementary figures and images for: Comprehensive analyses of single-cell and bulk RNA-seq reveal the biological and prognostic roles of BMP4 in pancreatic adenocarcinoma
Source: Front Mol Biosci. 2025 Oct 15;12:1686938. doi: 10.3389/fmolb.2025.1686938 (PMC12568421; doi:10.3389/fmolb.2025.1686938)

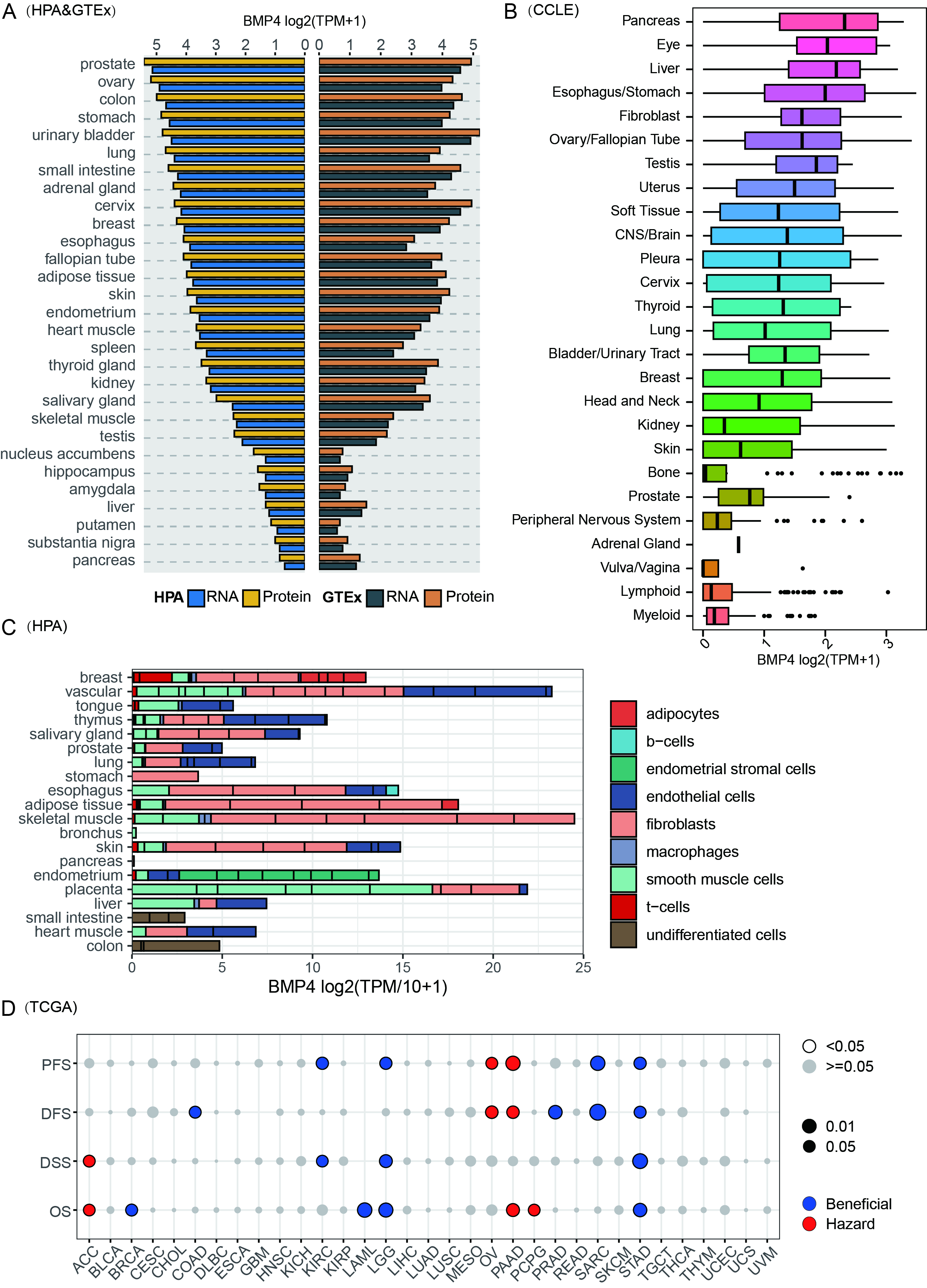

Supplement: Supplementary file 1 [file DataSheet1.zip › supplmentary materials/fig_s1.tif]

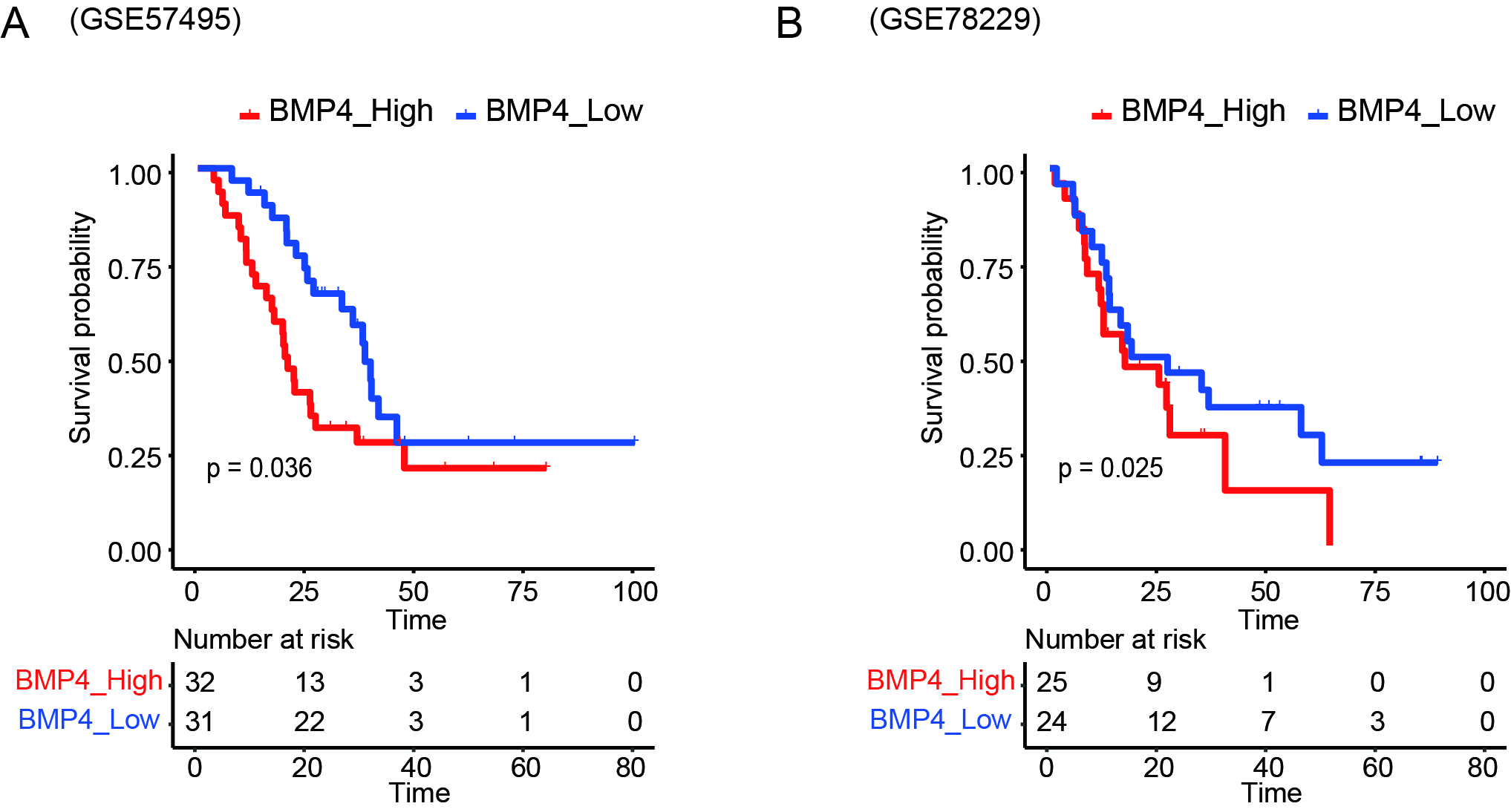

Supplement: Supplementary file 1 [file DataSheet1.zip › supplmentary materials/fig_s10.tif]

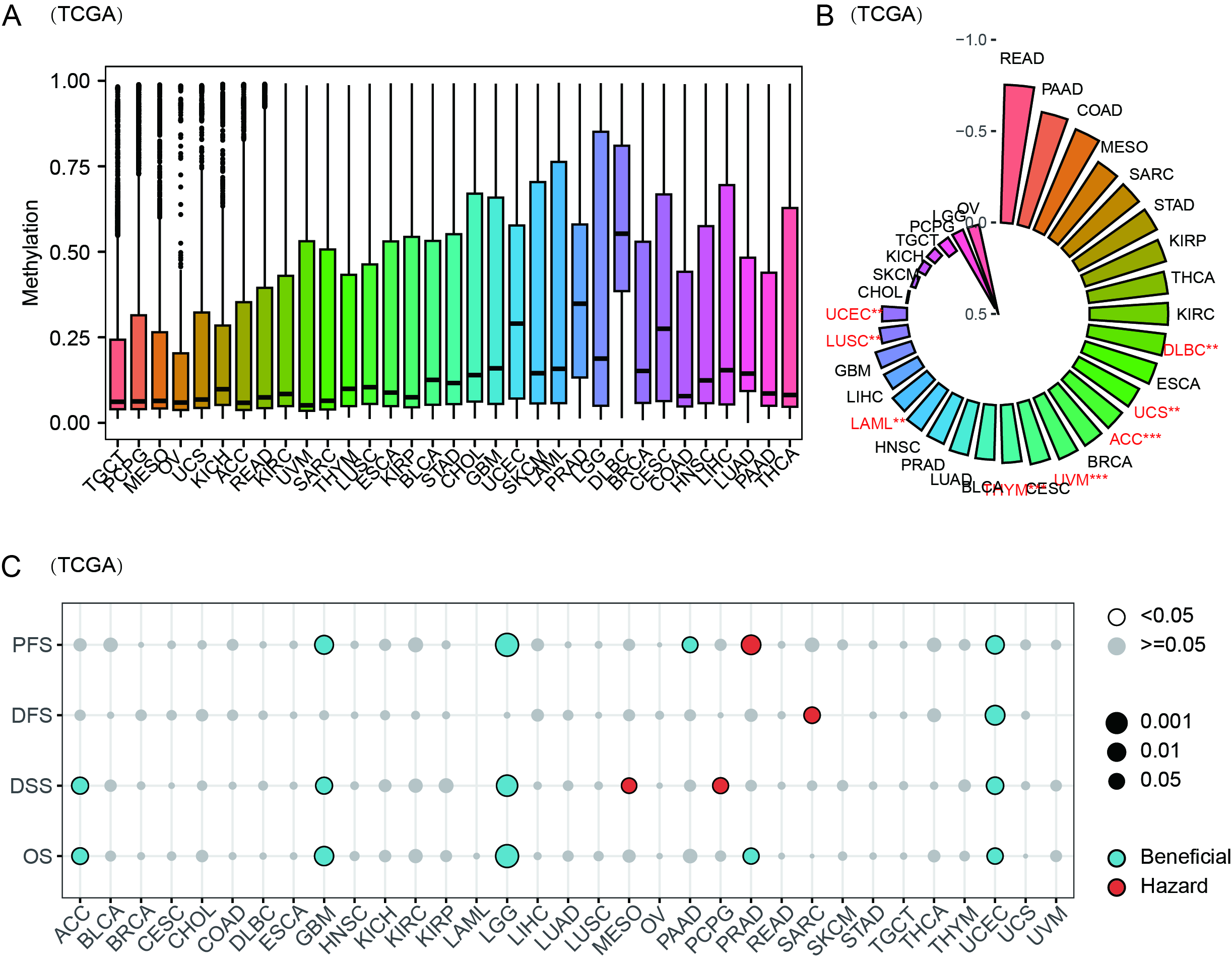

Supplement: Supplementary file 1 [file DataSheet1.zip › supplmentary materials/fig_s2.tif]

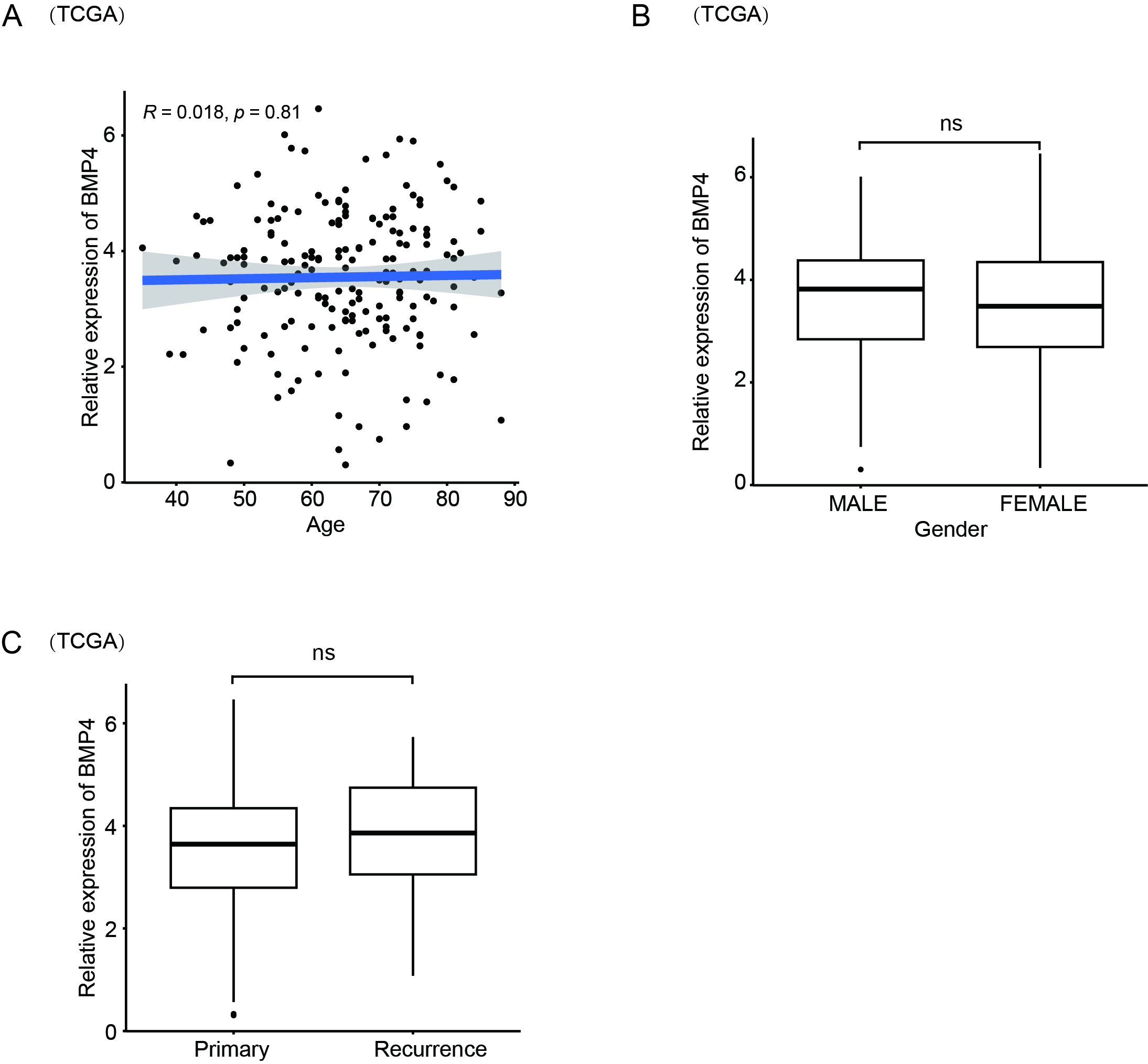

Supplement: Supplementary file 1 [file DataSheet1.zip › supplmentary materials/fig_s3.tif]

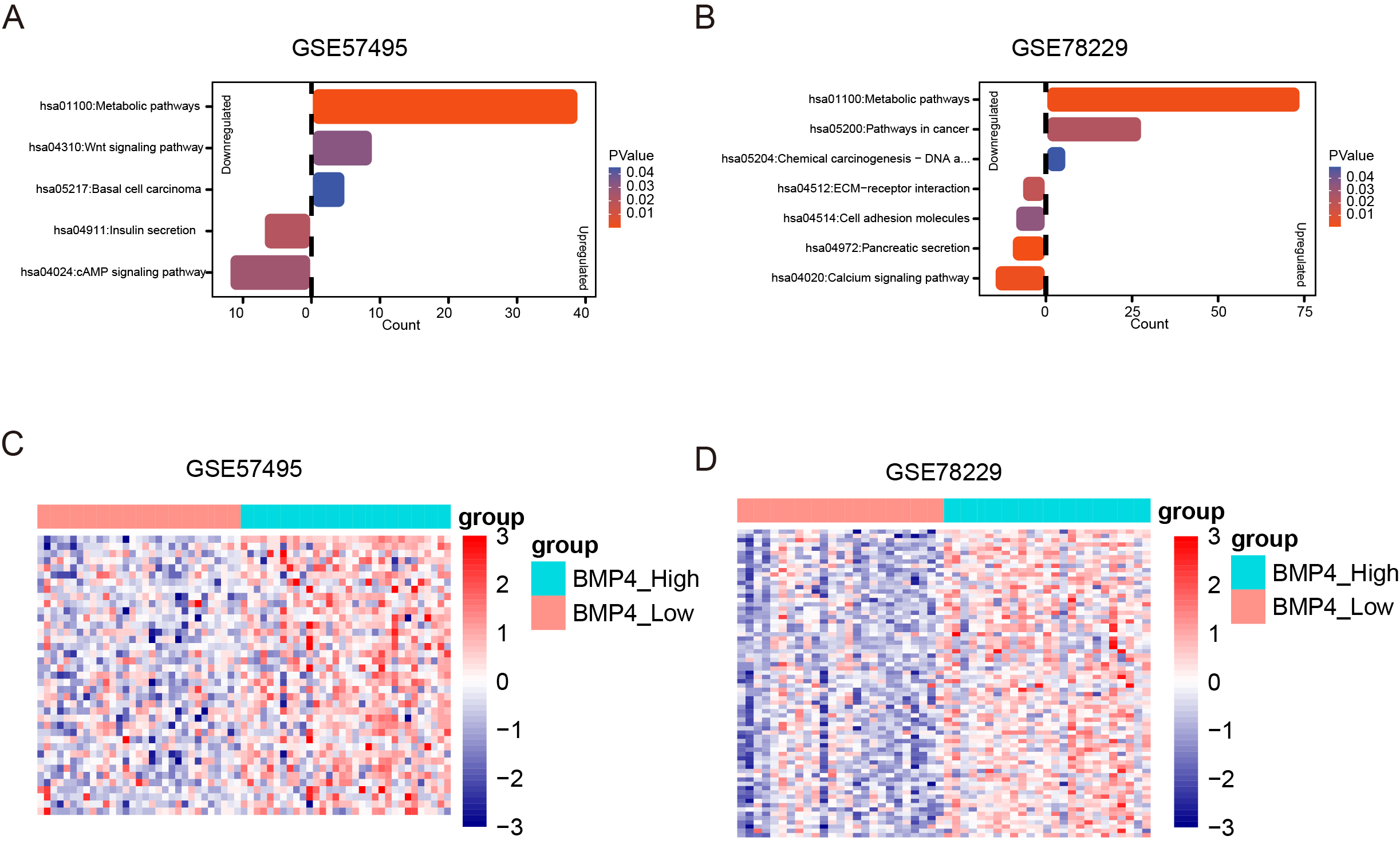

Supplement: Supplementary file 1 [file DataSheet1.zip › supplmentary materials/fig_s4.tif]

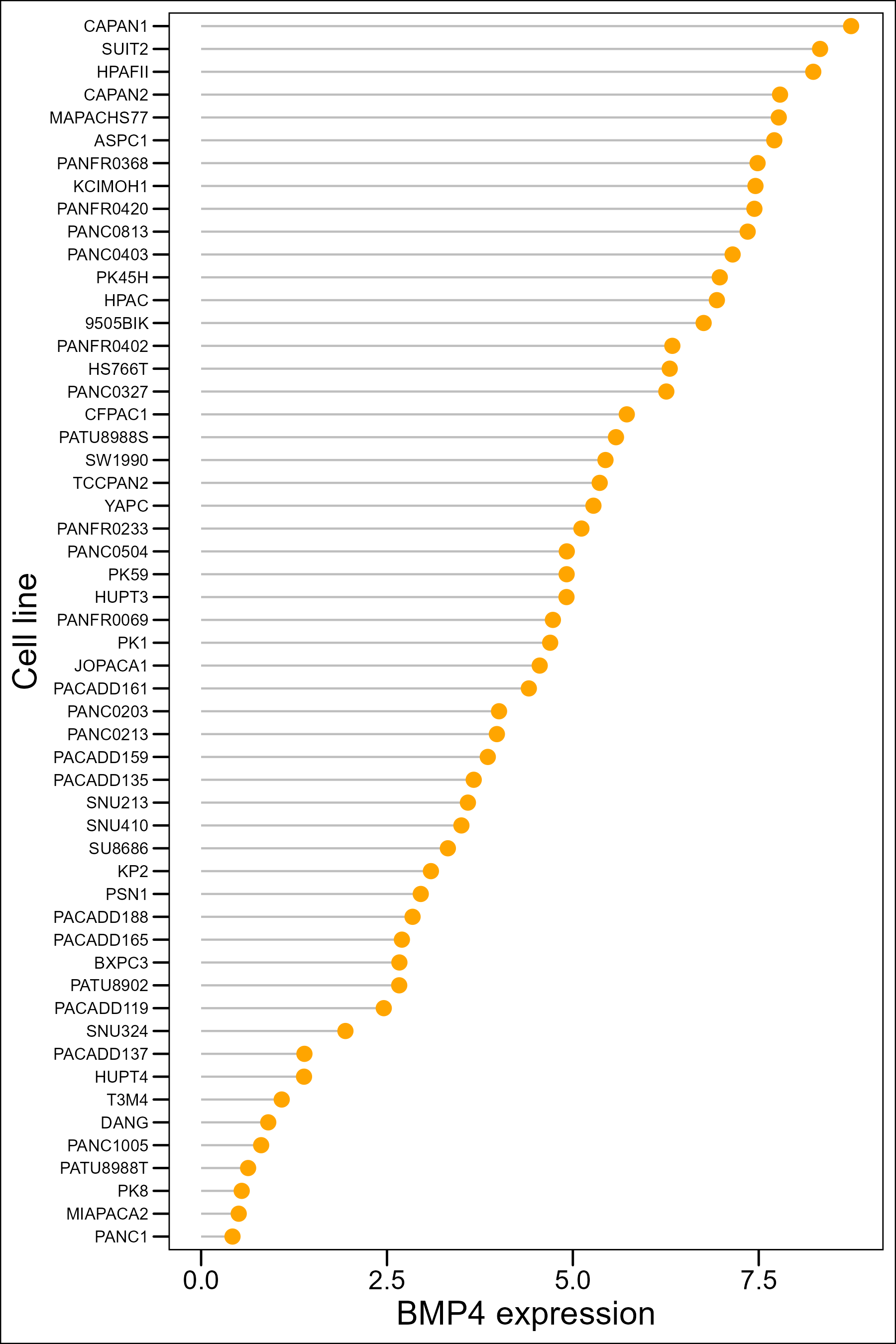

Supplement: Supplementary file 1 [file DataSheet1.zip › supplmentary materials/fig_s5.tiff]

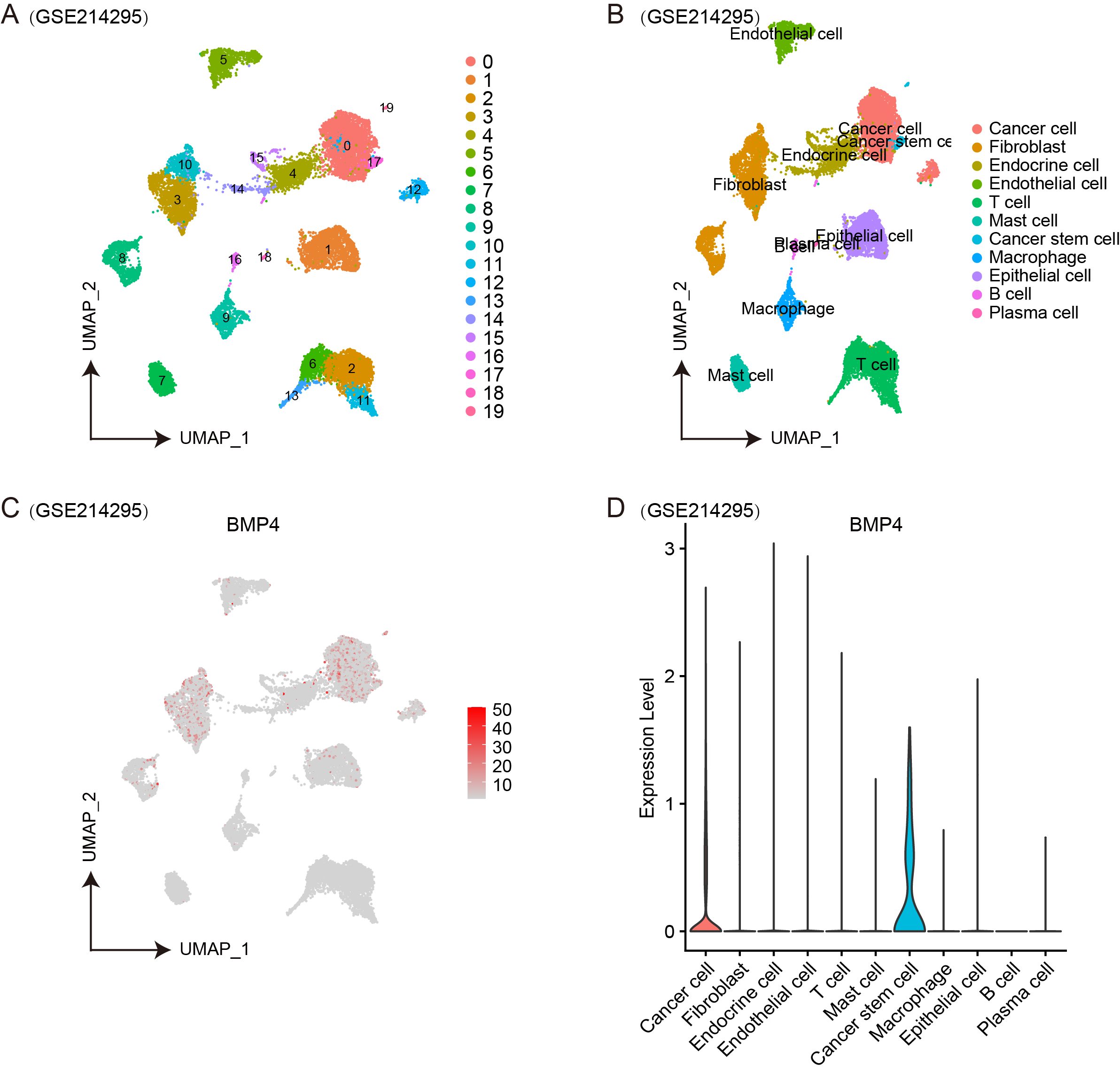

Supplement: Supplementary file 1 [file DataSheet1.zip › supplmentary materials/fig_s6.tif]

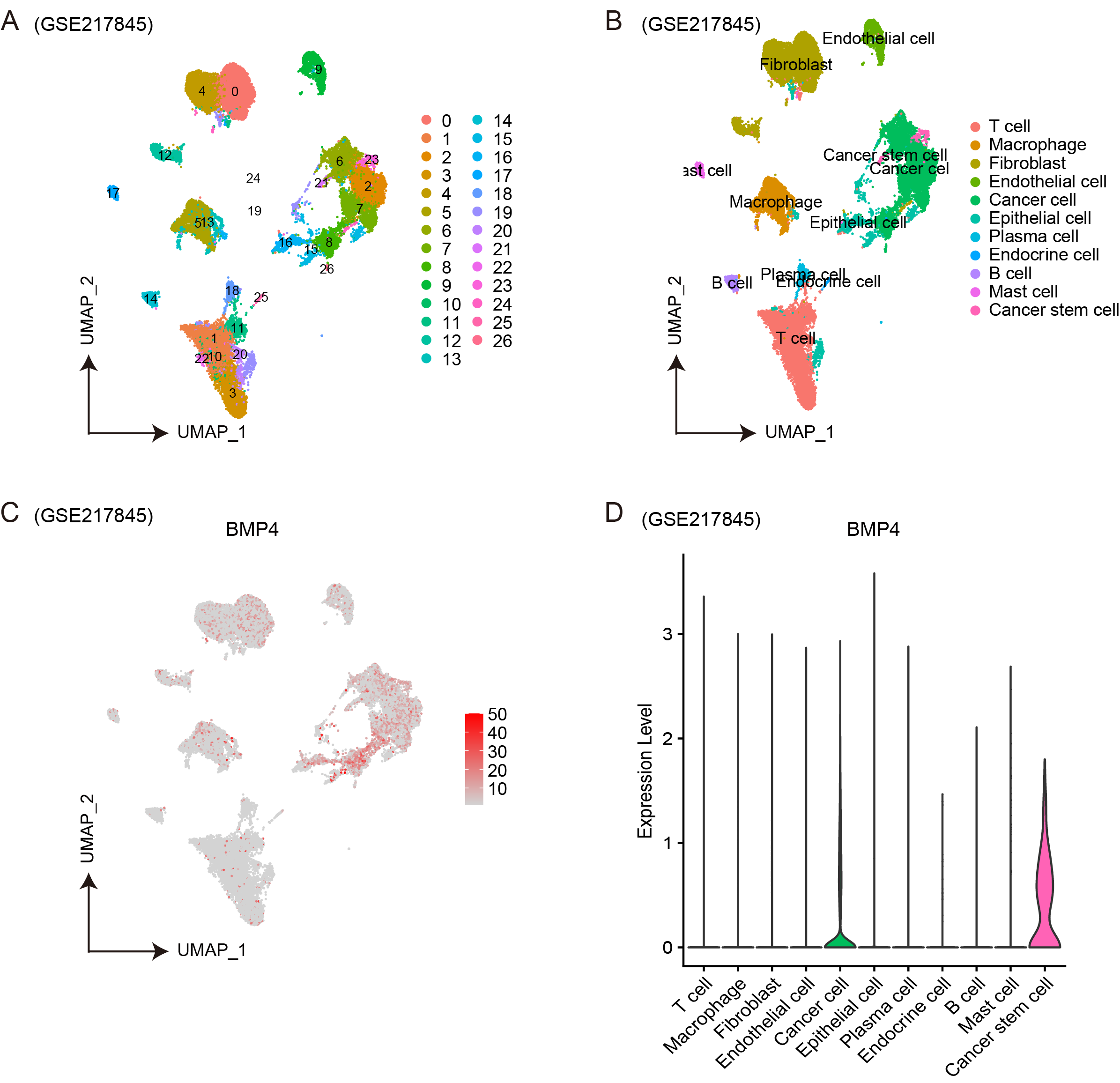

Supplement: Supplementary file 1 [file DataSheet1.zip › supplmentary materials/fig_s7.tif]

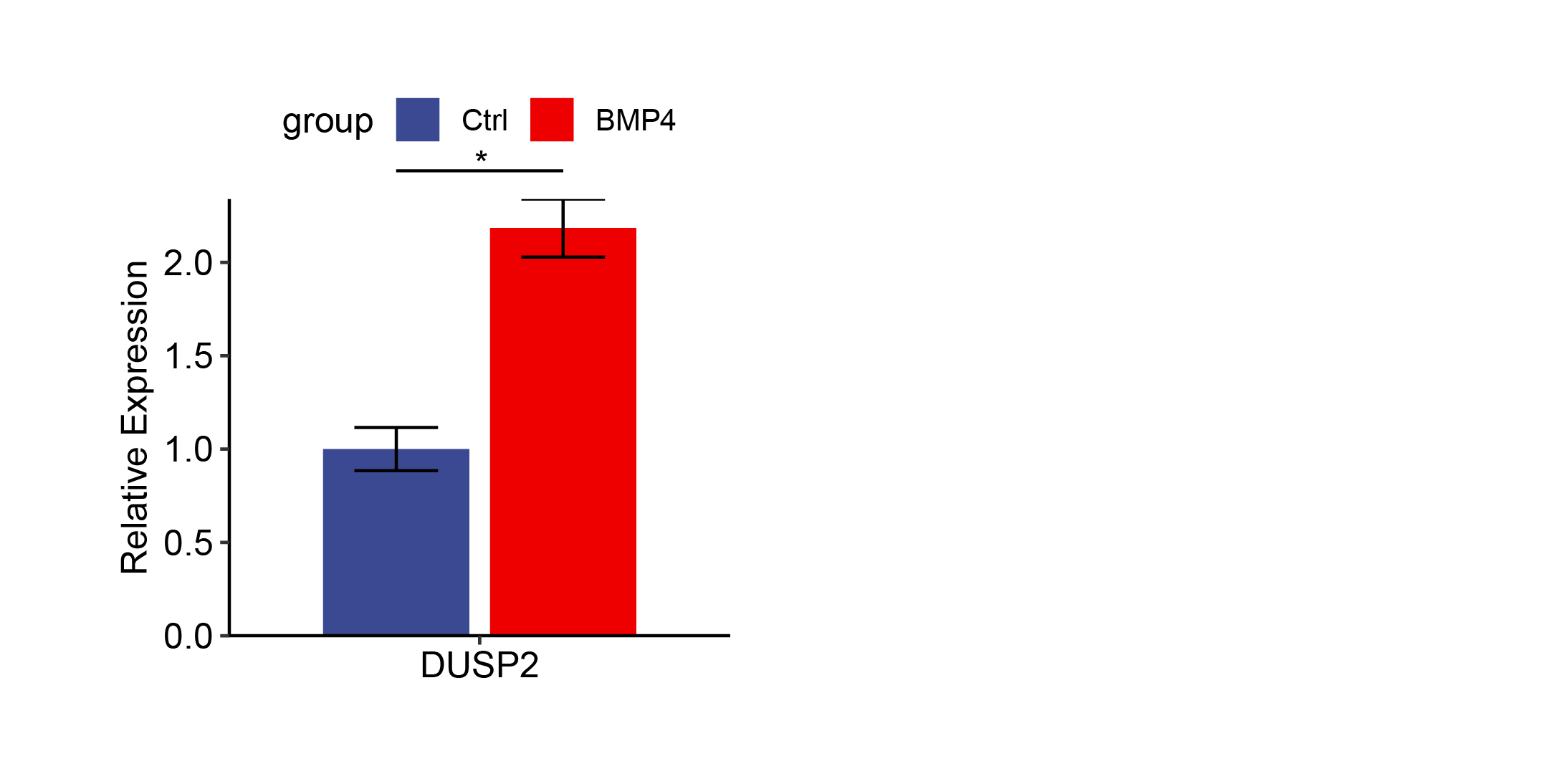

Supplement: Supplementary file 1 [file DataSheet1.zip › supplmentary materials/fig_s8.tif]

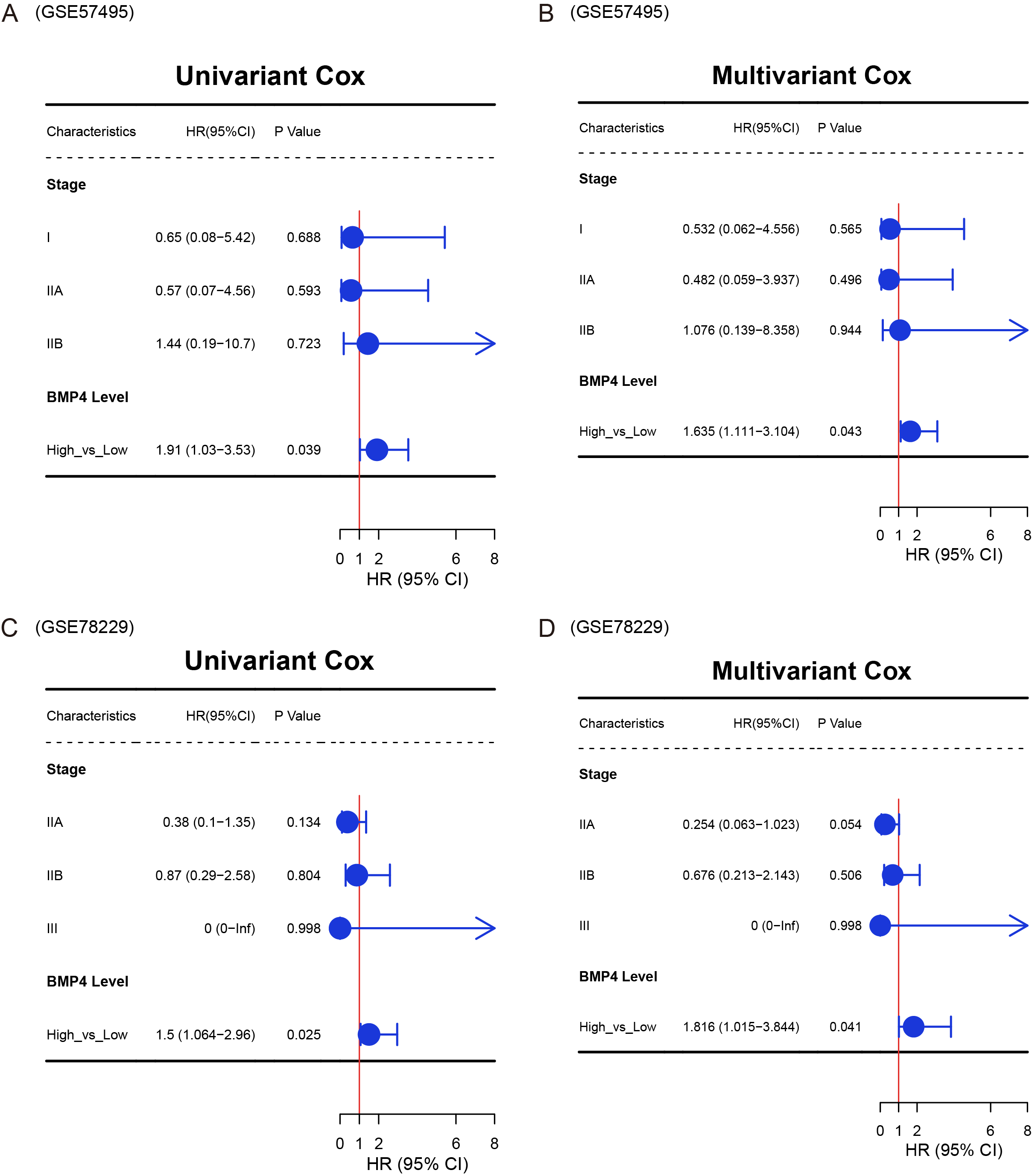

Supplement: Supplementary file 1 [file DataSheet1.zip › supplmentary materials/fig_s9.tif]
